# Performance Evaluation of RTS/CTS Scheme in Beacon-Enabled IEEE 802.15.6 MAC Protocol for Wireless Body Area Networks

**DOI:** 10.3390/s20082368

**Published:** 2020-04-22

**Authors:** Sana Ullah, Mohammad Mehedi Hassan, M. Shamim Hossain, Abdulhameed Alelaiwi

**Affiliations:** 1 Department of Computer and Software Technology, University of Swat, Swat 19130, Pakistan; sana@uswat.edu.pk; 2 Department of Information Systems, College of Computer and Information Sciences, King Saud University, Riyadh 11543, Saudi Arabia; 3 Department of Software Engineering, College of Computer and Information Sciences, King Saud University, Riyadh 11543, Saudi Arabia; mshossain@ksu.edu.sa (M.S.H.); aalelaiwi@ksu.edu.sa (A.A.)

**Keywords:** IEEE 802.15.6, RTS, CTS, wireless body area network, healthcare

## Abstract

The IEEE 802.15.6 standard has the potential to provide cost-effective and unobtrusive medical services to individuals with chronic health conditions. It is a low-power standard developed for wireless body area networks and enables wireless communication inside or near a human body. This standard utilizes a Carrier Sense Multiple Access with Collision Avoidance (CSMA/CA) protocol to improve network performance under different channel access priorities. However, the CSMA/CA proposed in the IEEE 802.15.6 standard has poor throughput performance and link reliability when some of the nodes deployed on a human body are hidden from each other. We employ the RTS/CTS scheme to solve hidden node problems in IEEE 802.15.6 networks over a lossy channel. To improve performance of the RTS/CTS scheme, we adjust transmission power levels of the nodes according to transmission failures. We estimate throughput and energy consumption of the proposed model by differentiating several parameters, such as contention window size, values of bit error ratios, number of nodes in different priority classes. The performance results are obtained through analytical approximations and simulations. We observe that the proposed model significantly improves performance of the IEEE 802.15.6 CSMA/CA by resolving hidden node problems.

## 1. Introduction

Heart disease is the leading cause of death worldwide. In 2016, 15.2 million people died as a result of ischemic heart disease and stroke globally [1]. In the same year, chronic obstructive pulmonary and lung diseases have killed 3 million and 1.7 million people, respectively, and while diabetes and dementia killed around 3.6 million people. At present, healthcare systems are facing major challenges due to rapid increase in human population, diseases, and health expenditure. According to [2], the healthcare cost in the United States was 17.9% of the total Gross Domestic Product (GDP) in 2017, and it is expected to reach 19.4% by 2027. Based on these statistics, the current healthcare systems require new cost-effective solutions. Wireless Body Area Network (WBAN) [3,4] has emerged as one of the key technologies to provide long term, remote, and cost-effective health monitoring of patients, thus detecting early signs of diseases and providing feedback in real time [5]. A WBAN is comprised of small and intelligent sensors that are implanted under the human skin or deployed on the human body to monitor vital signs of patients. Such sensors may be used to control several diseases such as heart disease, diabetes, obesity, and other chronic conditions [6]. For example, electrocardiogram patterns may be exploited to improve health status of patients suffering from ischemic heart disease [7]. With the inception of WBAN technology, researchers have focused on evaluating existing standards such as IEEE 802.15.4 [8] for WBAN. However, these standards have not been adapted due to the stringent quality of service requirements of nodes operating in close proximity to human tissues [9].

The IEEE has developed a new standard called IEEE 802.15.6 that allows wireless communication inside or near the human body, and satisfies the WBAN requirements [10]. This standard employs a Carrier Sense Multiple Access with Collision Avoidance (CSMA/CA) protocol that enables access to the channel based on different user priorities and contention window sizes. The current version of the IEEE 802.15.6 CSMA/CA encounters low throughput and high delay when some of the nodes are hidden or exposed. The hidden node problem occurs when a node deployed on the human body may communicate with the hub but may not be able to communicate with other nodes that are communicating with the same hub. The exposed node problem occurs when a node is unable to communicate with the hub due to interference with the neighboring transmission. The hidden and exposed node problems occur in a variety of scenarios including when multiple IEEE 802.15.6 networks sharing the same spectrum coexist with each other or when some of the nodes in a single IEEE 802.15.6 network are hidden or exposed due to body shadowing effects [11]. These problems may also occur when the nodes are deployed on different locations with non-line of sight communication. The RTS/CTS scheme has been proposed to address problems of hidden nodes in IEEE 802.11 and IEEE 802.15.4 networks. The RTS/CTS scheme reduces collisions when nodes are hidden from the hub. In case of problems associated with exposed node, it may increase throughput only when nodes are synchronized and are able to hear the CTS packets. As discussed in [11], the RTS/CTS scheme has to potential to significantly improve performance of the IEEE 802.15.6 CSMA/CA protocol.

### Contribution

In this paper, we utilize a power aware RTS/CTS scheme in the IEEE 802.15.6 CSMA/CA based networks in order to increase channel utilization when nodes are hidden in a network. The key contributions of this paper are:

- We employ RTS/CTS scheme to solve hidden node problems in IEEE 802.15.6 CSMA/CA based networks.
- We propose a power aware method that adjusts transmission power levels of nodes to avoid loss of RTS or CTS packets.
- We propose a mathematical model to validate the proposed approach.
- We perform extensive simulations to show effectiveness of the proposed approach over the conventional IEEE 802.15.6 CSMA/CA protocol in terms of throughput and energy consumption.

Under the assumptions of a finite number of nodes, we consider a lossy channel that may distort transmitting packets. When the RTS or CTS packets are lost, we adjust the transmission power levels of the nodes. This adjustment of transmission power levels is effective when the nodes intend to send high priority or life-critical data and emergency event reports to the hub. We analyze the performance of the proposed scheme using both analytical approximations and simulations. The results are derived for different priority classes as proposed in the IEEE 802.15.6 standard in terms of throughput and energy consumption. The results obtained in this paper may help us to understand different IEEE 802.15.6 network scenarios before deployment.

The rest of the paper is organized as follows. Section Two provides an overview of related work on the IEEE 802.15.6, while Section Three presents the RTS/CTS scheme in the IEEE 802.15.6 CSMA/CA protocol. Section Four presents analytical approximations and performance results, and Section Five concludes the paper.

## 2. Related Work

Previous studies have generally used analytical modeling to analyze the performance of the IEEE 802.15.6 CSMA/CA protocol. These models are based on the popular Bianchi’s model, which was proposed for the IEEE 802.11 standard [12]. Other models adapt extensive simulations to evaluate the performance of IEEE 802.15.6 contention-free protocols under different traffic scenarios. Furthermore, limited research work has been conducted on the utilization of the RTS/CTS scheme for this standard. We categorize extant literature related to our study in the following two subsections.

### 2.1. Analysis of IEEE 802.15.6 CSMA/CA

Several research studies on performance analysis of the IEEE 802.15.6 are based on Discrete Time Markov Chain (DTMC). Rashwand et al. proposed an early probabilistic model for this standard, which evaluates the effects of access phase periods over network performance [13]. By considering saturated and non-ideal channel conditions, they observed that the IEEE 802.15.6 CSMA/CA perform poorly for high traffic loads. The authors of [14] proposed a DTMC to evaluate throughput, delay and power consumption of IEEE 802.15.6 CSMA/CA for an error-prone channel. The proposed DTMC considers the delay of a node by taking into account the acknowledgement time after the packets are transmitted. Under a saturated traffic scenario, the authors analyzed the performance for different user priorities and concluded that five user priorities are enough to satisfy the network requirements. In another study [15], the authors proposed a Markov chain for all user priorities defined in the standard and concluded that high priority nodes have unfair access to the channel, thus degrading throughput of low priority nodes. The authors of [16] reached to the same conclusion for an ideal channel. They modeled the backoff process of the IEEE 802.15.6 CSMA/CA using a Markov chain, and analyzed the effects of traffic loads, modulation and coding, and different user priorities on saturated throughput and access delay. The author of [17] proposed an adaptive priority method that decreases the number of retransmission by considering the channel environment of IEEE 802.15.6 networks. Yuan et. al. improved the IEEE 802.15.6 standard by proposing an adaptive medium access control protocol [18]. The proposed protocol adjusts superframe and access phase lengths according to the nodes generating traffic. By considering three user priorities, the proposed protocol outperforms the IEEE 802.15.6 in terms of network delay and power consumption. In [19], the authors proposed a time-saturation model for IEEE 802.15.6 CSMA/CA by considering the effects of lost packets, which were ignored in the above-mentioned works. The authors observed that network throughput converges according to the system values, thus changing the time-saturation model to either saturated or non-saturation models. In [20], the authors introduced a novel and prioritized Fibonacci backoff scheme in IEEE 802.15.6 CSMA/CA to decrease node waiting time and number of collisions on the channel. By using a Markov chain, the authors observed that the proposed backoff scheme performs better than that of IEEE 802.15.6 in terms of throughput, power consumption, and head of line delay. The authors of [21] utilized a multi-beam directional antenna in the IEEE 802.15.6 CSMA/CA to extend network reliability and lifetime. In [22], the authors proposed a power-enabled priority scheme that reduces the number of retransmissions of packets. They concluded that the proposed scheme outperforms the IEEE 802.15.6 CSMA/CA in terms of bandwidth efficiency, energy consumption, throughput, and delay. The random transmission delay of the IEEE 802.15.6 CSMA/CA is analyzed in [23], where the authors used a probabilistic model for asynchronous duty-cycling networks. The authors concluded that the proposed model achieves better performance in terms of expectations and variance of the delay.

### 2.2. RTS/CTS Scheme for Previous Standards

The above studies have not considered hidden and exposed node problems that may degrade performance of the IEEE 802.15.6 networks for all user priorities. These problems have been solved for the previous standards including IEEE 802.11 and IEEE 802.15.4 using the RTS/CTS scheme. In [24], the authors studied the use of the RTS/CTS scheme to reduce the exposed node problem in IEEE 802.11 based wireless networks. By analyzing the signal strength of nodes in a 3D simulation environment, they concluded that the proposed scheme has effective throughput performance compared with that of the standard model. The authors of [25] improved the conventional RTS/CTS scheme proposed for IEEE 802.11 in order to classify why some transmission fail. According to the proposed model, the packets are immediately transmitted after failure due to noise. By adapting a four-dimensional Markov model, the authors observed that the proposed model enhances the performance in terms of average throughput and packet delay. As the RTS packets may collide during transmissions, the authors of [26] proposed an RTS collision avoidance method that decreases transmission cost and overhead of control packets compared to that of the IEEE 802.11 RTS/CTS scheme. In [27], the authors extended the conventional RTS/CTS scheme for multi-hop IEEE 802.15.4 networks. By proposing an adaptive RTS/CTS scheme, the authors concluded that adjusting RTS transmission according to the number of collisions achieves better performance. In a similar study, the authors of [28] analyzed the RTS/CTS scheme combined with packet concatenation for the non-beacon mode of IEEE 802.15.4 and concluded that the proposed scheme shows effective improvements in throughput, delay, and bandwidth efficiency. In [29], the authors studied different types of collisions in the presence of hidden and exposed nodes and analyzed undiscovered collisions between RTS and CTS packets for duty-cycled WBANs.

As discussed above, the RTS/CTS scheme is the most widely adapted scheme to solve most of the hidden node problems. However, this scheme has not been studied extensively for performance improvement of IEEE 802.15.6 CSMA/CA. In our work, we adapt the RTS/CTS scheme for this standard and provide a detailed analysis to validate our conclusions.

## 3. RTS/CTS Scheme for IEEE 802.15.6

The IEEE 802.15.6 standard organizes the nodes in a star topology network, where a single node (called the hub) controls operation and resource allocation in the network. This standard supports beacon and non-beacon enabled modes. In the latter, the hub may operate in (1) superframe-based allocations, where the hub utilizes a managed access phase defined in the standard for bilink allocation intervals, and (2) superframe-free based allocations, where the hub does not use superframe structures and adapts unscheduled type 2 allocations to send short and a fixed number of data packets. In the beacon-enabled mode, the hub employs multiple access protocols, such as slotted-ALOHA, CSMA/CA, and polling according to the traffic requirements. In this mode, the communication is bounded by superframe boundaries comprising many access phases such as random, exclusive, managed, and contention access phases. The most common access procedure used in the random-access phase is the IEEE 802.15.6 CSMA/CA protocol, because it supports eight priority levels according to the traffic classification. However, as mentioned above, this protocol encounters poor performance in the presence of hidden node problems.

### 3.1. Hidden Node Problem in IEEE 802.15.6

**Figure 1:**
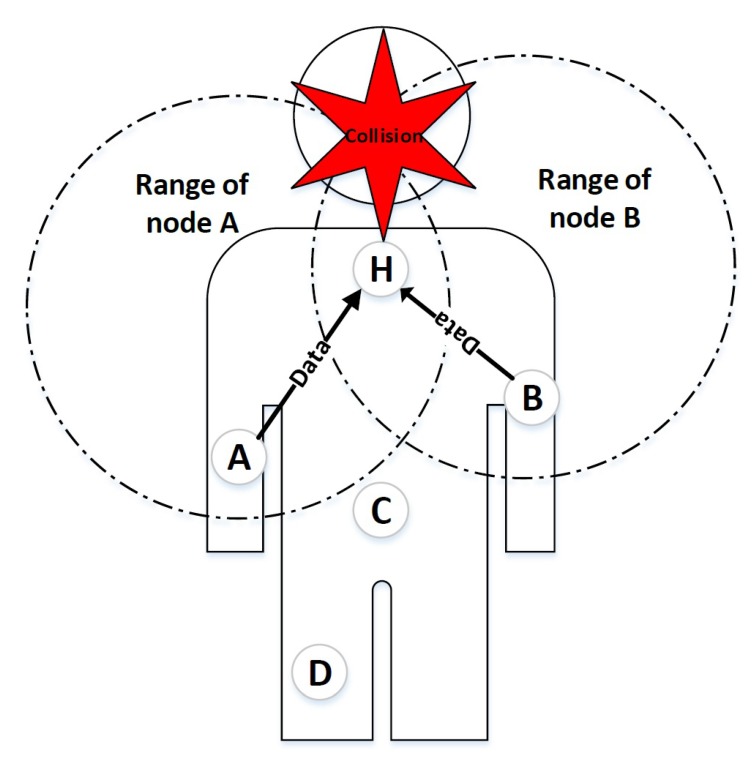
Hidden node problem in a single-hop IEEE 802.15.6 Carrier Sense Multiple Access with Collision Avoidance (CSMA/CA) based networks.

The hidden node problem occurs when some nodes are not in the wireless range of other nodes. In single-hop IEEE 802.15.6 CSMA/CA based networks, the hidden node problem may occur when some nodes deployed on the human body are in the range of the hub but not in the range of each other. This is possibly due to body shadowing effects or when the nodes are not in the line of sight, e.g., nodes deployed on the back of a human body may have a chance of becoming hidden nodes. Figure 1 shows a description of a hidden node in the IEEE 802.15.6 CSMA/CA based networks. As shown in this figure, node A is in the wireless range of the hub H1, and the same hub is in the wireless range of node B. However, node A and node B are not in each other’s wireless ranges and so are unable to sense their respective transmissions. Therefore, when node A transmits data to the hub, node B cannot hear this transmission and transmits a data packet to the same hub after sensing the channel. Since node B is not in the transmission range of node A, the channel is perceived to be free even though node A has already sent data to the hub. This scenario results in collision at the hub. The hidden node problem may also occur when multiple IEEE 802.15.6 based networks coexist with each other by sharing the same spectrum [11].

### 3.2. Exposed Node Problem in IEEE 802.15.6

**Figure 2:**
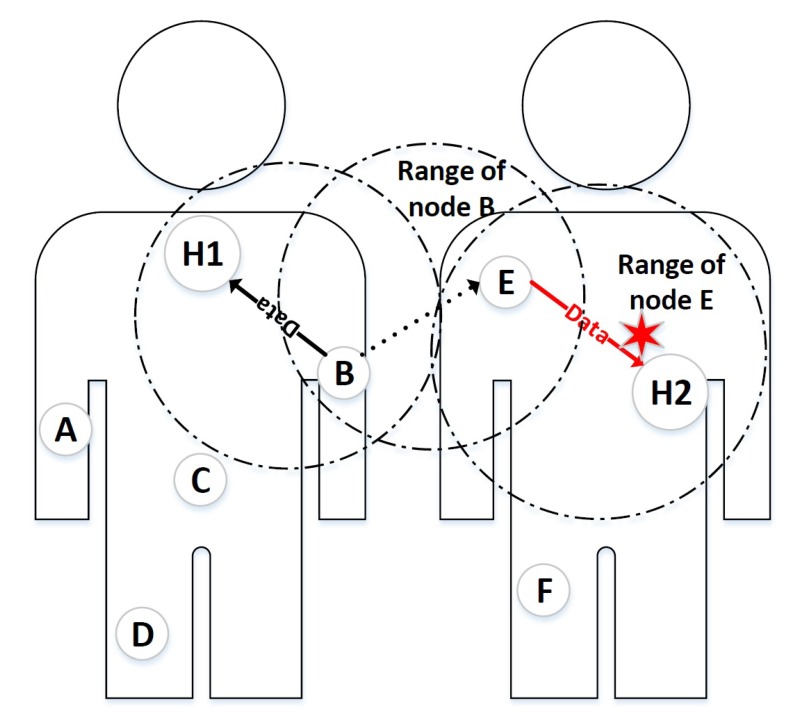
Exposed node problem in multiple IEEE 802.15.6 CSMA/CA based networks.

The exposed node problem occurs when some nodes are prevented from transmitting data due to interference with the neighboring transmission. This problem is likely to occur in inter-WBAN communication or when multiple networks coexist with each other. Figure 2 explains one of the possible scenarios of an exposed node problem in IEEE 802.15.6 CSMA/CA based networks. Both H1 and H2 are not in the transmission range of each other, however, the transmitting node B and node E of two adjacent networks are in the same transmitting range. When node B is sending data to H1, node E may defer its transmission to H2. However, node E could transmit data to H2 since both nodes are available for transmission. Unlike the hidden node problem that increases the number of collisions, the exposed node problem increases the delay of nodes by preventing them from transmitting data.

### 3.3. Proposed RTS/CTS Scheme

**Figure 3:**
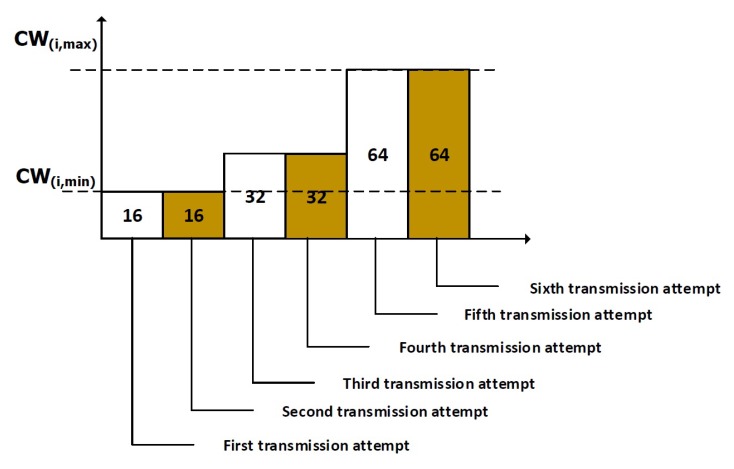
Backoff process in IEEE 802.15.6.

There are several methods to solve the above-mentioned problems in the existing literature, such as increasing transmission power levels of nodes to increase the wireless range of a network, using omni-directional antennas, moving the hidden nodes, and removing obstacles. As discussed above, most researchers have focused on adapting the RTS/CTS scheme to enhance the performance of the CSMA/CA protocol by resolving hidden and exposed node problems. The CSMA/CA defined in the IEEE 802.15.6 is different from the conventional CSMA/CA protocol because it adjusts the backoff process according to eight different user priorities. Unlike previous CSMA/CA, where the contention window size is doubled for each failure, the IEEE 802.15.6 CSMA/CA doubles the contention window size for an even number of failed attempts, as shown in Figure 3.

**Figure 4:**
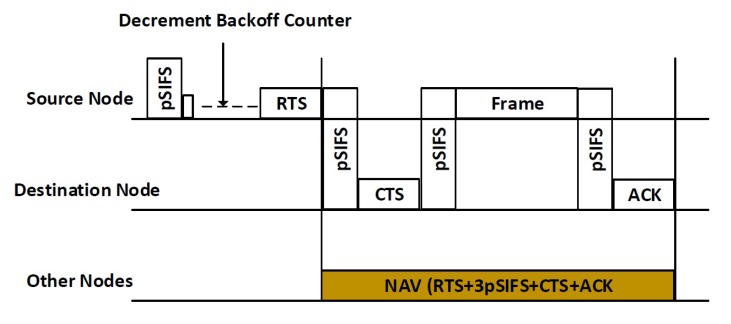
RTS/CTS scheme between two nodes in IEEE 802.15.6 CSMA/CA based networks.

In the proposed scheme, when the data frame is available for transmission, the node senses the channel to check ongoing activity or transmission by other nodes. When the channel is free for the duration of Short Interframe Spacing (called pSIFS in the standard), the node chooses a random number called a backoff counter in an interval (1, CW), where CW is the contention window. As shown in Algorithm 1, the $CW=CW_{i,min}$ in the first stage, where $CW_{i,min}$ indicates the minimum contention window for nodes in priority class *i*. The counter represented by *C* is decremented when the channel is free for the duration of a CSMA slot. When the counter reaches 0, the node transmits an RTS frame to the destination. When the CTS is received by the transmitter, the data frame is sent. Other nodes hold their transmission for the total transmission duration, or Network Allocation Vector (NAV). Figure 4 shows a general description of the RTS/CTS scheme between two nodes. If the frame collides in the channel due to simultaneous node transmission, the CW is doubled for an even number of failures. The value of CW may not exceed the maximum contention window represented by $CW_{i,max}$. The value of CW is not altered when the failure occurs for odd number of times. The backoff counter is frozen when the channel is busy or when the current time is outside the access phase lengths or when the there is not enough time left in the superframe to accommodate frame transmission.

When the node does not receive the CTS, it assumes that the RTS packet may have been lost, and therefore increases the transmission power level to the next level. This is shown in Algorithm 1, which is based on our previous work in [30]. The transmission power level represented by $P_{L}$ is incremented when the CTS is not received. We consider discrete power levels predefined in the transceiver. The discrete power levels will not exceed the maximum power level represented by $P_{Lmax}$. This technique extends the wireless range of nodes. It may help to solve the exposed node problem by including exposed nodes in the wireless range of the transmitter. It may be noted that increasing the transmission power of nodes affects overall power consumption, and therefore the power levels are increased for high priority nodes intending to send medical data. As shown in Algorithm 1, we consider priority class 5 where nodes send medical data to the hub. We assume that for patients with critical health status, reporting emergency events is most crucial, and this is achieved through an increase in the transmission power levels. This, however, does not largely affect the power consumption of nodes compared to the overall throughput obtained through such a power increase. The values *N*, $T_{sf}$, and $T_{f}$ represent the backoff stage, superframe duration, and frame duration, respectively.

**Algorithm 1** Proposed RTS/CTS scheme with power level adjustment for IEEE 802.15.6 CSMA/CA protocol.

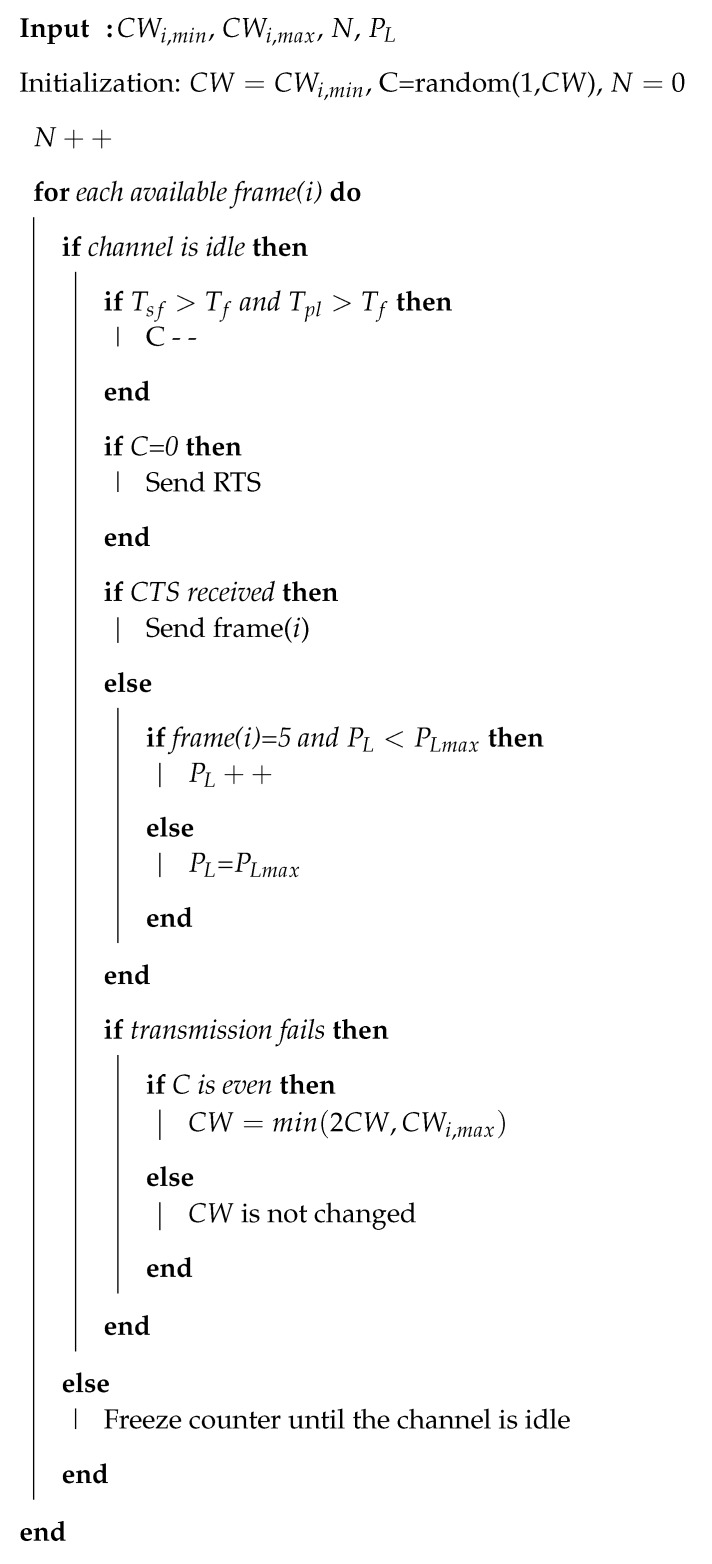



## 4. Performance Evaluation

### 4.1. Numerical Model

We focus on deriving throughput and energy consumption of the proposed model. We assume saturated and lossy channel conditions for all user priorities. As we assume a lossy channel, the control and data frames may be lost due to error or collisions on the channel. We assume three scenarios: (1) the frames collide on the channel, (2) the data packet including RTS, CTS, and ACK is corrupted due to error on the channel, and (3) the data is successfully transmitted.

Let $n_{i}$ be the finite number of nodes in priority class *i*. By following [31], the busy channel probability $p_{i}$ in the priority class *i* is given by

(1) $$p_{i}=b_{i}+\left(1-b_{i}\right)p_{r}$$

where $b_{i}$ and $p_{r}$ represent conditional collision and error probabilities, respectively. The channel is considered busy when other nodes transmit data. Our $p_{i}$ is different from [31] because it considers nodes of different user priorities.

Let $p_{e}$ be the average value of BER, we have $p_{r}=1-{\left(1-p_{e}\right)}^{T}$, where *T* is the total length of payload in bits and is calculated as

(2) $$\begin{array}{c} T=Preamble+PHY+MH+L+RTS+CTS+ACK \end{array}$$

where Preamble and PHY are the physical layer preamble and header, respectively. The MAC header is represented by MH, and the actual data is represented by *L*. The control packets RTS, CTS, and ACK indicate transmission of RTS, CTS, and acknowledgement packets, respectively.

The expression for $b_{i}$ is given by

(3) $$\begin{array}{c} b_{i}=1-{\left(1-\tau_{i}\right)}^{n_{i}-1}\prod_{j=0,j\neq i}^{7}{\left(1-\;\tau_{i}\right)}^{n_{j}} \end{array}$$

In Equation (3), $\tau_{i}$ represents the transmission probability of a node in the priority class *i*. We calculate the value of $\tau_{i}$ in the same manner as performed in our previous work in [32]. However, while calculating this value, we assume transmission of RTS and CTS packets. Once $\tau_{i}$ is known, we can find the probability of a successful transmission for priority class *i* using the following equation

(4) $$\begin{array}{c} s_{i}=n_{i}\tau_{i}{\left(1-\tau_{i}\right)}^{n_{i}-1}\prod_{j=0,j\neq i}^{7}{\left(1-\;\tau_{i}\right)}^{n_{j}} \end{array}$$

By following [12], the saturation throughput can be obtained as

(5) $$\begin{array}{c} S=\frac{s_{i}\left(1-p_{idle}\right)\left(1-p_{r}\right)T_{L}}{p_{idle}\rho +\left(1-pidle\right)\left(1-ps\right)T_{c}+p_{s}\left(1-p_{idle}\right)\left(1-{\left(1-p_{e}\right)}^{T}\right)T_{s}+p_{s}\left(1-p_{idle}\right){\left(1-p_{e}\right)}^{T}T_{s}} \end{array}$$

where $p_{idle}$ is the probability of an idle slot time and is given by

(6) $$\begin{array}{c} p_{idle}=\prod_{i=0}^{7}{\left(1-\;\tau_{i}\right)}^{n_{i}} \end{array}$$

In Equation (5), the term $s_{i}\left(1-p_{idle}\right)\left(1-p_{r}\right)$ represents successful transmission on a lossy channel for priority class *i*, where $1-p_{idle}$ represents at least one transmission on the channel. The term (1−pidle)(1−ps) represents collisions on the channel, while the term $p_{s}\left(1-p_{idle}\right)\left(1-{\left(1-p_{e}\right)}^{T}\right)$ represents an error in transmission. The final term $p_{s}\left(1-p_{idle}\right){\left(1-p_{e}\right)}^{T}$ represents successful transmission in a slot time. The term $T_{L}$ is actual data transmission time. The term $T_{c}$ represents data collision or when no CTS is received due to collision. The term $T_{s}$ represents successful data transmission. To consider an error on the channel, we assume the worst scenario where the acknowledgement packet is lost after sending all preceding packets successfully. Therefore, the term $p_{s}\left(1-p_{idle}\right)\left(1-{\left(1-p_{es}\right)}^{T}\right)T_{s}$ in the above equation represents that the last packet, i.e., the acknowledgement, is lost. However, since it has already been transmitted, its value cannot be ignored in our calculations. The values of $T_{c}$ and $T_{s}$ are as follows

(7) $$\begin{array}{c} T_{c}=T_{RTS}+2pSIFS+\rho +\psi \end{array}$$

(8) $$\begin{array}{c} T_{s}=T_{H}+T_{RTS}+T_{CTS}+4pSIFS+T_{ACK}+\rho +\psi \end{array}$$

where ρ and ψ indicate backoff slot time and propagation delay, respectively. In Equation (7), the node waits for the duration of two pSIFS duration, i.e., one for resuming the backoff counter and another for sending the RTS packet.

To calculate the energy consumption, we assume that the major sources of energy wastage are collisions, successful transmission, and error on the channel. The total energy consumption is given by

(9) $$\begin{array}{c} E_{i}=\bar{P_{tx}}\left(1-p_{i}^{k+1}\right)\left(T_{H}+T_{RTS}+T_{L}\right)+P_{rx}\left(4pSIFS+T_{ACK}\right)+\\ P_{rx}\frac{EB_{i}}{1-b_{i}}b_{i}T_{1}+P_{rx}\frac{p_{s}p_{r}}{\left(1-pidle\right)}T_{1} \end{array}$$

where the first and second terms indicate energy consumption during successful transmission after k+1 attempts. As the proposed scheme assumes multiple power transmission levels, we consider the average transmitting power represented by $\bar{P_{tx}}$, which is the average value of transmitting power $P_{tx}$ at different power levels. The term $T_{H}$ is the transmission time of physical layer preamble, header, and MAC header. The term $P_{rx}$ represents power consumption in the receiving state. The value of $P_{rx}$ is not incremented. The third term in Equation (9) indicates energy consumption when the channel is busy, where $E(B_{i})$ is the mean backoff delay for nodes in the priority class *i*. The value of $E(B_{i})$ is determined by following [32]. The last term indicates energy consumption owing to error on the channel.

### 4.2. Simulation Environment

For the performance analysis, we consider a star topology network where the nodes send data to the hub over a lossy channel under saturated traffic scenarios. Initially, we consider 10 nodes in the network; however, the values of throughput and energy consumption are presented against several nodes in priority class 5, class 3, and class 1. The sizes of the minimum and maximum contention windows are taken according to [10]. For priority class 5, class 3, and class 1, the values of $\left(CW_{i,min},CW_{i},max\right)$ are set to (4,8), (8, 16), and (16, 32). The different values of contention windows allow nodes to obtain priority access to the channel depending on their traffic type. We assume a heterogeneous network with multiple nodes priorities. The operation of the proposed protocol is implemented in an independent C++ simulator by closely following all details and parameters of IEEE 802.15.6. The size of the data packet is 1560 bits. The narrow band physical layer considered in our analysis is 2400 MHz to 2483.5 MHz with 971.4 kbps data rate. The physical layer preamble and header are transmitted at a symbol rate (600 ksps) and a header rate (91.9 kbps) in the aforementioned physical layer, while the data packet is transmitted at 971.4 kbps. The size of RTS, CTS, and ACK packets is 193 bits. Table 1 shows values of all parameters used to derive the results. According to the IEEE 802.15.6, the value of ρ is calculated as ρ = CCA time + 40 μs, where CCA time is given by 63/symbol rate. We assume five power levels for high priority nodes, i.e., the power is incremented five times only. As we consider a lossy channel, the results are derived for BER = $10^{-5}$ and BER = $10^{-4}$.

### 4.3. Results

#### 4.3.1. Throughput

**Figure 5:**
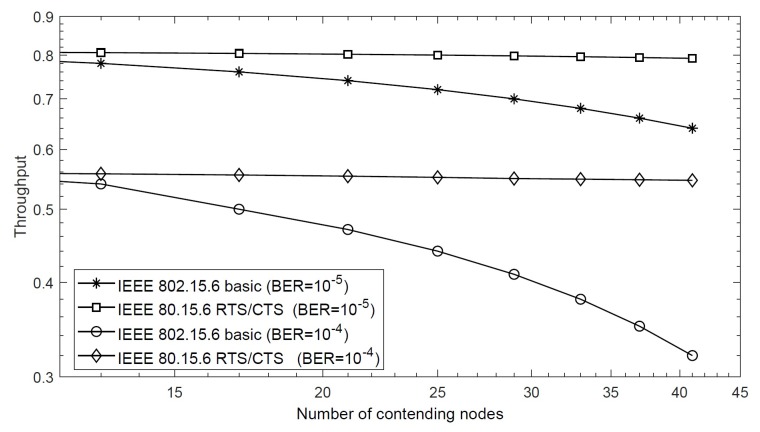
Saturation throughput of the RTS/CTS scheme for IEEE 802.15.6.

Figure 5 shows saturation throughput of the proposed scheme for a different number of nodes in class 5. For BER = $10^{-5}$ and BER = $10^{-4}$, it is observed that the use of the RTS/CTS scheme significantly increases overall throughput when compared with the basic IEEE 802.15.6 CSMA/CA protocol. However, the throughput is decreased both for the RTS/CTS scheme and basic protocol when we increase the value of BER. For example, for 17 nodes, the saturation throughput of the proposed RTS/CTS scheme is 0.80 when the value of BER = $10^{-5}$; however, the same protocol achieves 0.55 saturation throughput for BER = $10^{-4}$. We also observe in the figure that the performance of the IEEE 802.15.6 CSMA/CA decreases when the number of nodes (including hidden nodes) contending for the medium increases. Unlike the RTS/CTS scheme, where only the RTS packet is transmitted after winning access to the channel, the IEEE 802.15.6 CSMA/CA transmits the data packet, which decreases channel utilization and throughput in case of collisions, or when hidden nodes are present in the network. The throughput of the IEEE 802.15.6 CSMA/CA depends on the network size, while the throughput achieved by the RTS/CTS scheme is less sensitive to network size. This shows that employing RTS/CTS scheme is efficient and scalable for larger networks.

**Figure 6:**
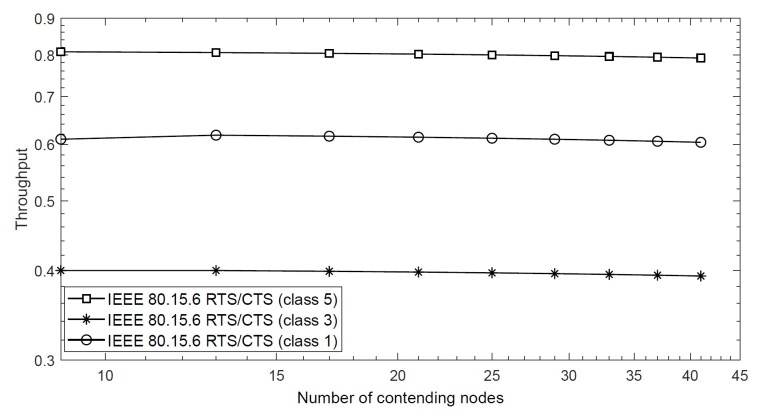
Saturation throughput of the RTS/CTS scheme for different classes with BER = $10^{-5}$.

**Figure 7:**
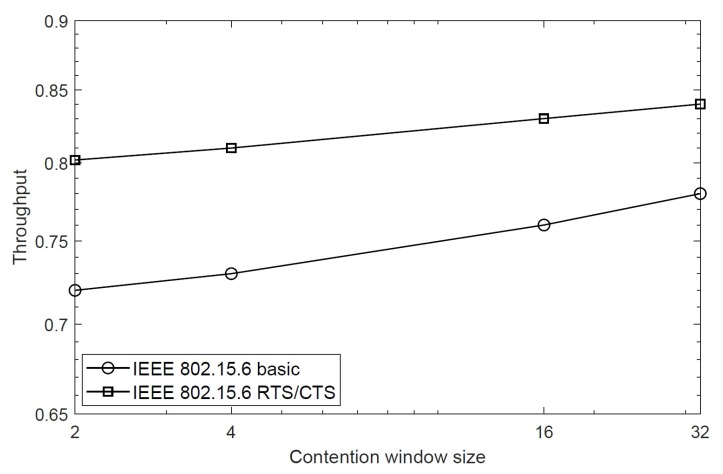
Saturation throughput of the RTS/CTS scheme for different contention window sizes.

However, when we analyze the saturation throughput of the RTS/CTS scheme for different priority classes, we note that high priority classes result in higher throughput due to small contention window size. This is because the RTS packets are immediately transmitted by high priority nodes, and hence, they achieve quick access to the channel. This is shown in Figure 6, where the proposed scheme achieves 0.8 saturation throughput for 15 nodes in class 5 with BER = $10^{-5}$, while the same scheme achieves 0.6 and 0.4 throughput values for class 3 and class 1, respectively. It is important to explain that the IEEE 802.15.6 CSMA/CA also achieves the same throughput trend for different priority classes, as explained in our previous work [32]. We found that it achieves higher throughput for high priority classes due to small backoff duration; however, the difference is that the throughput decreases as a function of network size, as discussed above. The effect of contention window size on the saturation throughput is presented in Figure 7. This figure considers nodes of the same priority with the same contention window sizes in a network. For BER = $10^{-5}$, the figure shows that overall throughput of the proposed scheme largely depends on the size of the contention window of the same priority nodes, i.e., larger contention window size is able to accommodate a large number of nodes, thus increasing the throughput. When the value CW is 16, the RTS/CTS scheme obtains 0.85 throughput compared with the basic scheme, which is 0.75. The afore-mentioned results show that the RTS/CTS scheme achieves significant throughput when compared with the conventional IEEE 802.15.6 CSMA/CA protocol for different BER values and number of nodes.

#### 4.3.2. Energy Consumption

**Figure 8:**
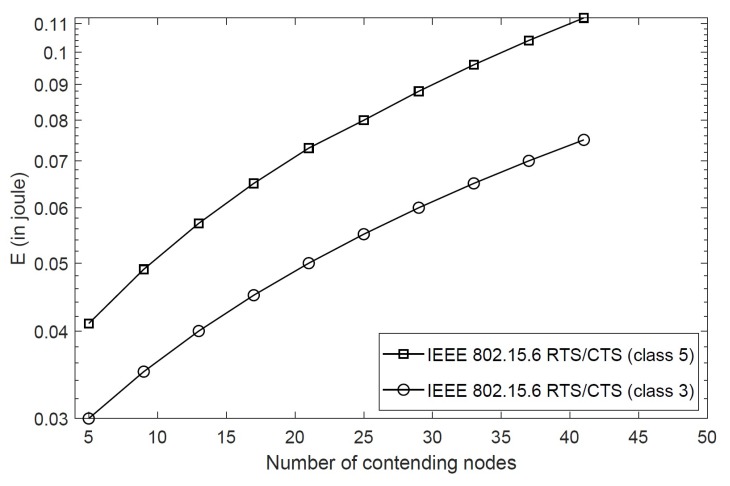
Energy consumption of the RTS/CTS scheme for different classes.

**Figure 9:**
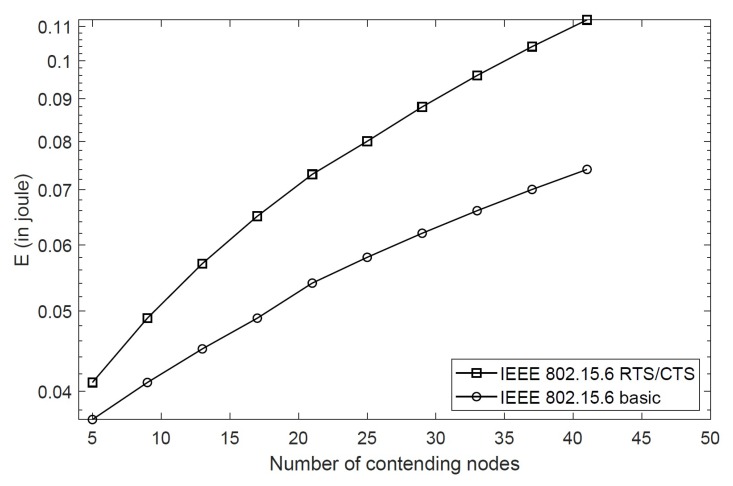
Energy consumption of the RTS/CTS scheme and IEEE 802.15.6 CSMA/CA.

Figure 8 presents the energy consumption of different nodes in class 5 and class 3 with BER = $10^{-5}$. As discussed above, high priority classes achieve higher throughput, which eventually results in higher energy consumption as shown in the figure. The proposed scheme consumes 0.04 joules energy for five nodes in class 5, and this consumption is increased to 0.11 joules for 40 nodes in the same class. This is due to collisions caused by the increasing number of nodes required to send RTS packets. The nodes in class 3 consume less energy as they stay in the backoff stage in the presence of high priority nodes. Figure 9 compares the energy consumption of the proposed scheme with the IEEE 802.15.6 CSMA/CA for different nodes in class 5 with BER = $10^{-5}$. As the RTS/CTS packets are exchanged in addition to data packets, they consume extra energy due to control packet overhead, especially when the size of packets are small. However, as reporting medical data or emergency events are extremely important in WBANs, it is of relevance that the RTS/CTS scheme is able to achieve maximum throughput in such situations, and therefore, the additional energy consumption is tolerable for life-critical applications. For example, the IEEE 802.15.6 CSMA/CA consumes less energy, but it is unable to report emergency events in the presence of hidden nodes or when the emergency events are generated at such nodes. The proposed scheme achieves better performance and is able to report emergency events generated at hidden nodes successfully.

## 5. Conclusions and Future Works

### 5.1. Conclusions

We proposed the RTS/CTS scheme for IEEE 802.15.6 CSMA/CA based networks that enhance overall performance in the presence of hidden nodes. We considered different transmission power levels to extend the transmission range of nodes in case of CTS failure. By deriving analytical expressions, we analyzed the throughput and energy consumption of the proposed scheme for different priority classes and BER values and concluded that the RTS/CTS scheme is able to achieve best performance when compared with the basic IEEE 802.15.6 CSMA/CA protocol. The results presented in this paper may provide guidelines to select optimal parameters and protocols when deploying WBANs for different healthcare scenarios. The proposed scheme has the potential to solve exposed node problems by extending the transmission range of exposed nodes.

### 5.2. Future Work

In the future, we will provide a detailed numerical and simulation-based analysis of the non-contention based protocols proposed in the IEEE 802.15.6 standard. Other research works in this direction are

- Research work on the development of energy efficient routing protocols over the IEEE 802.15.6 standard is required to enable communication between multiple WBANs.
- Further research at MAC layer is required to study exposed node problems when multiple WBANs coexist.
- Performance evaluation of scheduled-based mechanisms used in the IEEE 802.15.6 standard may be studied for different medical applications.
- The effects of adjusting transmission power levels of nodes in the RTS/CTS based IEEE 802.15.6 networks may be extensively studied in the future.

## Figures and Tables

**Table 1:** Standard parameters for 2400 MHz-2483.5 MHz.

Parameter	Values	Parameter	Values
RTS	193 bits	CTS	193 bits
Preamble	90 bits	PHY	31 bits
MH	72 bits	ψ	1 μs
Initial $P_{tx}$	33 mW	$P_{rx}$	8 mW
pSIFS	75 μs	*L*	1560 bits
ACK	193 bits	Power levels	5
